# Coexistent cerebral small vessel disease and multiple infarctions predict recurrent stroke

**DOI:** 10.1007/s10072-022-06027-6

**Published:** 2022-04-01

**Authors:** Yu Tian, Yuesong Pan, Hongyi Yan, Xia Meng, XingQuan Zhao, Liping Liu, Yongjun Wang, Yilong Wang

**Affiliations:** 1 Department of Neurology, Beijing Tiantan Hospital, Capital Medical University, No 119 South 4th Ring West Road, Fengtai District, Beijing, 100070 China; 2 Chinese Institute for Brain Research, No 119 South 4th Ring West Road, Fengtai District, Beijing, 100070 China; 3 China National Clinical Research Center for Neurological Diseases, No 119 South 4th Ring West Road, Fengtai District, Beijing, 100070 China; 4 Advanced Innovation Center for Human Brain Protection, Capital Medical University, No 119 South 4th Ring West Road, Fengtai District, Beijing, 100070 China; 5 Beijing Key Laboratory of Translational Medicine for Cerebrovascular Disease, No 119 South 4th Ring West Road, Fengtai District, Beijing, 100070 China

**Keywords:** Ischaemic stroke, TIA, Recurrent stroke, Cerebral small vascular disease, Infarction number

## Abstract

**Background and purpose:**

To investigate the association of different status of cerebral small vessel disease (CSVD) and infarction number with recurrence after acute minor stroke and transient ischaemic attack (TIA).

**Methods:**

This study was a post hoc analysis of the Clopidogrel in High-risk Patients with Acute Nondisabling Cerebrovascular Events (CHANCE) trial, and includes 886 patients with acute minor stroke and TIA. The status of CSVD and infarction number was recorded for each individual. Infarction number were classified as multiple acute infarctions (MAIs≥2), single acute infarction (SAI =1), and non-acute infarction (NAI =0). The CSVD burden were grouped into non-CSVD (0 score) and CSVD (1–4 score). The primary outcome was a recurrent stroke at the 1-year follow-up. The secondary outcomes were recurrent ischaemic stroke, composite vascular event (CVE), and TIA. We analyzed the relationships between different status of CSVD burden and infarction pattern with the risk of outcomes using multivariable Cox regression models.

**Results:**

Among all 886 patients included in present analysis, recurrent stroke was occurred in 93 (10.5%) patients during 1-year follow-up. After adjusted for all potential covariates, compared with patients with non-CSVD and NAI, patients with CSVD and MAIs were associated with approximately 9.5-fold increased risk of recurrent stroke at 1 year (HR 9.560, 95% CI 1.273–71.787, *p*=0.028). Similar results observed in ischaemic stroke and CVE.

**Conclusion:**

The status of CSVD and infarction number predicted recurrent stroke in patients with acute minor stroke and TIA, especially for those with coexistent CSVD and MAIs.

**Supplementary Information:**

The online version contains supplementary material available at 10.1007/s10072-022-06027-6.

## Introduction

Minor ischaemic stroke(MIS) and transient ischemic attack (TIA) are two common manifestations of cerebrovascular disease with mild or transient symptoms and non-disabling consequences [1]. However, despite owing to the implementation of standardized treatment, a part of patients after MIS/TIA still occurred subsequent stroke events, especially in the early period [2–4]. As secondary stroke was often more severe and disabling than the index event, early identification and risk stratification were of the utmost significance in preventing recurrence in patients with acute minor stroke or TIA.

Nowadays, baseline neuroimaging features, such as infarction number [5], cerebral small vessel disease(CSVD), and [6] intracranial atherosclerosis (ICAS) [7], were well-known crucial parameters for predicting stroke recurrence after MIS/TIA. Patients with multiple acute infarctions (MAIs), usually caused by cardiogenic embolism, artery-to-artery embolism, and other embolisms from undetermined sources, had a higher risk of recurrent stroke than patients with single acute infarction (SAI) or non-acute infarction (NAI) [5, 8]. CSVD, as a common intrinsic cerebral microvascular pathology, was the main cause of acute lacunar stroke and also increased the incidence of secondary stroke [9, 10]. The presence of ICAS, which could induce atherosclerotic plaques or thrombus formation, resulted in higher rates of new stroke events in TIA and minor stroke [7, 11]. Magnetic resonance imaging (MRI) markers on the baseline, which implied underlying mechanisms and pathologies of acute ischaemic stroke, were used to stratify the risk of recurrent events.

Furthermore, the combination of different neuroimaging parameters may provide more prognostic information than them only. For instance, a previous study showed a combined effect of the presence of both MAIs and ICAS of at least 1 of intracranial arterial segments on the risk of recurrent stroke in patients with minor stroke or TIA. [12] However, the coexistent CSVD and ICAS on baseline MRI were not associated with an increased risk of any recurrent stroke [13]. Our previous analysis of the CHANCE trial (the Clopidogrel in High-risk Patients with Acute Nondisabling Cerebrovascular Events trial) showed the copresence of CSVD and ICAS did not increase the risk of new strokes and disability in patients with minor stroke or TIA [14].

It highlighted that baseline neuroimaging signs of CSVD burden and infarction patterns may be two distinct aspects of underlying vascular mechanisms of stroke and TIA [15–18]. Although both of them were identified as predictors for recurrent events, there was no previous evidence that the combination of CSVD burden and infarction patterns could improve the efficiency of risk stratification in patients with acute minor stroke or TIA.

Using data from the Clopidogrel in High-Risk Patients with Acute Non-disabling Cerebrovascular Events (CHANCE) trial, we aimed to investigate the association between the neuroimaging status of CSVD burden and infarction pattern and recurrent stroke during 1-year follow-up, illustrating the potential combined influence of them on outcome in patient with MIS and TIA.

## Methods

### Overview of the CHANCE trial and the imaging substudy

The CHANCE trial was a randomized, multicentric, double-blind, placebo-controlled clinical study in China from October 2009 to July 2012. Details about the rationale, design, and results of the CHANCE trial have been previously published [19, 20]. Patients who completed MRI examinations (3.0 or 1.5 Tesla) at baseline were included in the imaging subgroup [12, 14, 21]. MR sequences included T1-weighted imaging, T2-weighted imaging, fluid-attenuated inversion recovery, diffusion-weighted imaging (DWI), and three-dimensional time-of-flight MR angiography. In the present post hoc analysis, we derived data from the prespecified imaging subgroup of the CHANCE trial. Patients without MRI examination or with incomplete sequences to assess CSVD and infarction number were excluded in this analysis.

### Data availability statement

The CHANCE trial registered on clinicaltrials.gov (NCT00979589). Ethics approval was obtained by the ethics committee of Beijing Tiantan Hospital (IRB approval number: KY2015-001-01) and all participating centers. All participants provided written informed consent before inclusion into the study. The data are available from the corresponding author upon reasonable request.

### Neuroimage analyses

MR images were collected from participating centers in digital format and were evaluated by two senior neurologists in Beijing Tiantan Hospital. MRI analysts are blinded to clinical characteristics and outcomes.

According to the number of acute ischaemic lesions, the infarction pattern was stratified as MAIs, SAI, and NAI [22, 23]. NAI was defined as no hyperintense lesion on DWI. SAI was defined as an uninterrupted hyperintense lesion on DWI visible in contiguous territories, while MAIs were defined as more than one hyperintense lesions on DWI that were separated in space or discrete on contiguous slices.

According to the STandards for ReportIng Vascular changes on nEuroimaging [24], MRI markers of CSVD, including white matter hyperintensity(WMH), lacuna, microbleeds (CMBs), and enlarged perivascular space (ePVS), were calculated as the total CSVD burden score ranged from 0 to 4 [6]. One point was allocated to each of the following markers: (1) severe periventricular WMH (Fazekas grade 3) or moderate to severe deep WMH (Fazekas grade 2–3); (2) presence of lacuna; (3) presence of microbleed; (4) moderate to severe (>10) basal ganglia ePVS. According to the total burden of CSVD, patients were categorized into two groups: non-CSVD group (score 0) and CSVD group (score 1–4). Patients with CSVD further divided into slight CSVD (score 1–2) and severe CSVD (score 3–4) [14].

### Follow-up and study outcome assessment

In the present analysis, the efficacy outcomes were analogous with those of the CHANCE trial, except the outcomes were extended to 1-year follow-up period. The primary efficacy outcome was a recurrent stroke, including ischaemic stroke and hemorrhagic stroke, and secondary efficacy outcomes included recurrent ischaemic stroke, composite vascular event (CVE), TIA. The definitions of stroke, ischaemic stroke, CVE, and TIA were in accord with previously reported outcomes of the CHANCE trial [19].

### Statistical analysis

Baseline characteristics were compared among patients with different statuses of CSVD burden and infarction pattern. Categorical variables were presented as percentages and the *χ*^2^ test was performed for categorical variables; continuous variables were presented as mean with standard deviation (SD) or median with interquartile range (IQR), and one-way analysis of variance or Kruskal-Wallis test was adopted for continuous variables.

In the primary analysis, the associations between different CSVD burden and varying infarction number with recurrent events were assessed using Cox regression models. Hazard ratios (HRs) and 95% CIs were calculated based on two models. In model 1, we only adjusted for age and sex; in model 2, we adjusted for age, gender plus the potential covariates(including body mass index(BMI), history of ischaemic stroke, TIA, coronary artery disease, atrial fibrillation, hypertension, diabetes, hypercholesterolemia, smoking status, time to randomization, qualifying event, NIHSS score on admission, and antiplatelet therapy). The time to the new stroke events of each group was presented by the Kaplan- Meier curves.

In a secondary analysis, we tested for interaction and stratified analysis of infarction pattern with CSVD in determining the risk of stroke recurrence, and we also conducted a sensitivity analysis in which the association between randomized antiplatelet therapies on recurrent events in patients with different MR phenotypes of CSVD was investigated.

The level of significance was *p*<0.05, and all tests were 2-sided. All analyses were performed with the SAS statistical software, version 9.4 (SAS Institute, Cary, North Carolina, USA).

## Results

### Demographics and baseline characteristics

From October 2009 to July 2012, a total of 5,170 patients were enrolled in the CHANCE trial, and 1,089 patients among them were recruited in the imaging subgroup. Finally, a number of 886 patients were included in the present analysis. Clinical characteristics of included and excluded in all patients of the CHANCE trial were shown in Supplement Table 1, and baseline characteristics of included patients and those of excluded in imaging subgroup were shown in Supplement Table 2.

Among the 886 patients in this analysis, 58(6.5%) patients had non-CSVD with NAI, 136(15.3%) patients had slight CSVD with NAI, 89(10.0%) patients had severe CSVD with NAI, 86(9.7%) patients had non-CSVD with SAI, 171(19.3%) patients had slight CSVD with SAI, 141(15.9%) patients had severe CSVD with SAI, 50(5.6%) patients had non-CSVD with MAI, 92(10.0%) patients had slight CSVD with MAI, and 63(7.1%) patients had severe CSVD with MAI. Demographics and baseline characteristics of patients with different statuses of total CSVD burden and infarction number were shown in Table 1. Compared with those with non-CSVD and NAI, patients with more CSVD burden and infarction number tended to be elder, have higher NIHSS score on admission, have more history of hypertension, congestive heart failure, and ischaemic stroke, and have more minor stroke as the qualifying event than TIA.

**Table 1:** Baseline characteristics according to CSVD and infarction number

Characteristics	No CSVD with NAI (*n*=58)	Slight CSVD with NAI (*n*=136)	Severe CSVD with NAI (*n*=89)	No CSVD with SAI (*n*=86)	Slight CSVD with SAI (*n*=171)	Severe CSVD with SAI (*n*=141)	No CSVD with MAIs (*n*=50)	Slight CSVD with MAIs (*n*=92)	Severe CSVD with MAIs (*n*=63)	***p*** value
Age, years, median(IQR)	56.5(48.1–62.3)	63.2(56.4–71.5)	69.2(63.0–74.9)	57.1(50.3–63.1)	59.4(52.7–68.3)	67.7(61.1–73.5)	58.6(51.7–67.5)	63.6(55.5–74.2)	68.2(60.0–74.2)	<0.001
BMI, kg/m^2^, median (IQR)	24.6(22.4–26.8)	24.1(22.0–26.7)	24.2(22.5–25.7)	24.3(23.0–26.4)	24.8(22.9–26.2)	24.2(22.1–26.0)	25.1(22.9–26.7)	23.9(21.3–25.9)	24.2(21.8–26.6)	0.23
Sex, male (%)	34(58.6)	82(60.3)	67(75.3)	59(68.6)	114(66.7)	97(68.8)	35(70.0)	65(70.7)	37(58.7)	0.25
Alcohol use, *n* (%)	20(34.5)	38(27.9)	30(33.7)	28(32.6)	63(36.8)	37(26.2)	20(40.0)	32(34.8)	14(22.2)	0.27
Tobacco use, *n* (%)	25(43.1)	55(40.4)	33(37.1)	43(50.0)	78(45.6)	58(41.1)	24(48.00)	48(52.2)	24(38.1)	0.43
History of disease, *n* (%)										
Ischaemic stroke	2(3.5)	20(14.7)	20(22.5)	8(9.3)	24(14.0)	36(25.5)	7(14.0)	15(16.3)	14(22.2)	0.002
TIA	3(5.2)	2(1.5)	0(0)	1(1.2)	3(1.8)	4(2.8)	1(2.0)	4(4.4)	3(4.8)	0.37
Myocardial infarction	1(1.7)	3(2.2)	4(4.5)	0(0)	2(1.2)	4(2.8)	2(4.00)	0(0)	3(4.8)	0.25
Congestive heart failure	1(1.7)	3(2.2)	1(1.1)	1(1.2)	1(0.6)	0(0)	2(4.0)	2(2.2)	5(7.9)	0.01
Atrial fibrillation	1(1.7)	5(3.7)	3(3.4)	1(1.2)	1(0.6)	4(2.8)	1(2.0)	3(3.3)	2(3.2)	0.75
Diabetes mellitus	8(13.8)	20(14.7)	17(19.1)	15(17.4)	47(27.5)	28(19.9)	13(26.0)	20(21.7)	15(23.8)	0.17
Hypertension	25(43.1)	92(67.6)	66(74.2)	39(45.6)	112(65.5)	103(73.1)	29(58.0)	53(57.6)	50(79.8)	<0.001
Hyperlipidemia	7(12.1)	19(14.0)	9(10.1)	9(10.5)	28(16.4)	11(7.8)	5(10.0)	11(12.0)	11(17.5)	0.43
ICAS	30(51.7)	74(54.8)	42(47.2)	35(40.7)	72(42.1)	65(46.1)	40(80.0)	63(68.5)	38(60.3)	<0.001
NIHSS score on admission, median(IQR)	1(0–2)	1(0–2)	2(1–2)	2(1–3)	2(1–3)	2(1–3)	2(1–3)	2(1–3)	2(1–3)	<0.001
Mean time to randomization, hour, median(IQR)	13.4(8.0–19.7)	11.1(7.6–19.0)	15.0(8.8–20.5)	11.4(7.0–18.5)	13.0(8.5–20.0)	13.3(9.0–21.0)	10.0(6.6–17.3)	11.8(6.8–20.3)	12.3(7.6–20.5)	0.19
Qualifying event, *N* (%)										<0.001
Minor stroke	40(69.0)	94(69.1)	80(90.0)	78(90.7)	157(91.8)	132(93.6)	42(84.0)	81(88.0)	56(88.9)	
TIA	18(31.0)	42(30.9)	9(10.1)	8(9.3)	14(8.2)	9(6.4)	8(16.0)	11(12.0)	7(11.1)	
Antiplatelet therapy, *n* (%)										0.81
Aspirin only	28(48.3)	66(48.5)	50(56.2)	40(46.5)	95(55.6)	71(50.4)	27(54.0)	43(46.7)	31(49.2)	
Clopidogrel+aspirin	30(51.7)	70(51.5)	39(43.8)	46(53.5)	76(44.4)	70(49.6)	23(46.0)	49(53.3)	32(50.8)	

*BMI*, body mass index; *TIA*, transient ischaemic attack; *NIHSS*, National Institutes of Health Stroke Scale; *ICAS*, Intracranial atherosclerotic stenosis; *CSVD*, cerebral small vessel disease; *NAI*, non-acute infarction; *SAI*, single acute infarction; *MAIs*, multiple acute infarctions.

When grouped by infarction number, patients with CSVD were older than those without CSVD (Table 2). In the NAI group, patients with CSVD are more likely to be with a history of ischaemic stroke, TIA, and hypertension. Patients with both SAI and CSVD had more prior ischaemic stroke and hypertension and longer time to randomization than those with SAI and non-CSVD. In the MAIs group, patients with CSVD were more likely to have a history of ICAS and lower BMI.

**Table 2:** Baseline characteristics according to the presence of CSVD when grouped by infarction number

Characteristics	NAI(*n*=283)			SAI(*n*=398)			MAIs(*n*=205)		
	Non-CSVD (*n*=58)	CSVD (*n*=225)	*p* value	Non-CSVD (*n*=86)	CSVD (*n*=312)	*p* value	Non-CSVD (*n*=50)	CSVD (*n*=155)	*p* value
Age, years, median(IQR)	56.5(48.1–62.3)	66.1(59.5–73.3)	<0.001	57.1(50.3–63.1)	62.9(55.3–71.2)	<0.001	58.6(51.7–67.5)	64.9(56.8–74.2)	<0.001
BMI, kg/m^2^, median (IQR)	24.6(22.4–26.8)	24.2(22.0–26.3)	0.26	24.3(23.0–26.4)	24.5(22.5–26.1)	0.50	25.1(22.9–26.7)	24.1(21.5–26.0)	0.04
Sex, male (%)	34(66.2)	149(66.2)	0.28	59(68.6)	211(67.6)	0.86	35(70.7)	102(65.8)	0.58
Alcohol use, *n* (%)	20(34.5)	68(30.2)	0.53	28(32.6)	100(32.1)	0.93	20(40.0)	46(29.7)	0.17
Tobacco use, *n* (%)	25(43.1)	88(39.1)	0.58	43(50.0)	136(43.6)	0.29	24(48.0)	72(46.5)	0.85
History of disease, *n* (%)									
Ischaemic stroke	2(3.5)	40(17.8)	0.006	8(9.3)	60(19.2)	0.03	7(14.0)	29(18.7)	0.45
TIA	3(5.2)	2(0.89)	0.03	1(1.2)	7(2.2)	0.53	1(2.0)	7(4.5)	0.42
Myocardial infarction	1(1.7)	7(3.1)	0.57	0(0)	6(1.9)	0.20	2(4.0)	3(1.9)	0.41
Congestive heart failure	1(1.7)	4(1.8)	0.98	1(1.2)	1(0.3)	0.33	2(4.0)	7(4.5)	0.88
Atrial fibrillation	1(1.7)	8(3.6)	0.48	1(1.2)	5(1.6)	0.77	1(2.0)	5(3.2)	0.65
Diabetes mellitus	8(13.8)	37(16.4)	0.62	15(17.4)	75(24.0)	0.20	13(26.0)	35(22.6)	0.62
Hypertension	25(43.1)	158(70.2)	0.001	39(45.4)	215(68.9)	<0.001	29(58.0)	103(66.5)	0.28
Hyperlipidemia	7(12.1)	28(12.4)	0.94	9(10.5)	39(12.5)	0.61	5(10.0)	22(14.2)	0.45
ICAS	30(51.7)	116(51.8)	0.99	35(340.7)	137(43.9)	0.59	40(80.0)	101(65.2)	0.05
NIHSS score on admission, median(IQR)	1(0–1)	1(0–2)	0.45	2(1–3)	2(1–3)	0.37	2(1–3)	2(1–3)	0.17
Mean time to randomisation, hour, median(IQR)	13.4(8.0–20.0)	12.0(8.0–20.0)	0.81	11.4(7.0–18.5)	13.0(8.5–12.1)	0.04	10.0(6.6–17.3)	12.2(7.0–20.5)	0.22
Qualifying event, *N* (%)			0.19			0.55			0.42
Minor stroke	40(69.0)	18(31.0)		78(90.7)	289(92.6)		42(84.0)	137(88.4)	
TIA	174(77.3)	51(22.7)		8(9.3)	23(7.4)		8(16.0)	18(11.6)	
Antiplatelet therapy, *n* (%)			0.66			0.27			0.44
Aspirin only	28(48.3)	116(51.6)		40(46.5)	166(53.2)		27(54.0)	74(47.7)	
Clopidogrel+aspirin	30(51.7)	109(48.4)		46(53.4)	146(46.8)		23(46.0)	81(52.3)	

*BMI*, body mass index; *TIA*, transient ischaemic attack; *NIHSS*, National Institutes of Health Stroke Scale; *ICAS*, Intracranial atherosclerotic stenosis; *CSVD*, cerebral small vessel disease; *NAI*, non-acute infarction; *SAI*, single acute infarction; *MAIs*, multiple acute infarctions.

### Outcomes by CSVD and infarction number respectively

Overall, 93(10.5%) patients in this study had new stroke at 1-year follow-up. Among them, 8(8.6%), 51(54.8%), 34(36.6%) of individuals had NAI, SAI, and MAIs, respectively (Table 3). Both the presence of SAI (HR 4.65, 95% CI 2.17–10.00, *p*<0.001, Model 1; HR 5.92, 95% CI 2.70–13.00, *p*<0.001, Model 2) and MAIs (HR 4.88, 95% CI 2.31–10.29, *p*<0.001, Model 1; HR 6.34, 95% CI 2.94–12.70, *p*<0.001, Model 2) were associated with an increased risk of new stroke both in Model 1 adjusted for age and sex and in Model 2 adjusted for age, sex and other potential covariates. Similar results were observed in new ischaemic stroke and CVE.

**Table 3:** Risk of outcomes by presence/total burden of CSVD in patients with minor ischaemic stroke or TIA at 1-year follow-up

Outcomes		No.	Event, *n* (%)	Model 1		Model 2	
				Adjusted HR(95%CI)	*p* value	Adjusted HR(95%CI)	*p* value
Stroke							
Infarction number	NAI	283	8(2.8)	Ref.		Ref.	
	SAI	398	51(12.1)	4.88(2.31–10.29)	<0.001	4.65(2.17–10.00)	<0.001
	MAI	205	34(16.6)	6.34(2.94–13.70)	<0.001	5.92(2.70–13.00)	<0.001
Total CSVD burden	0	194	19(9.8)	Ref.		Ref.	
	1–2	399	50(12.5)	1.24(0.72–2.13)	0.43	1.17(0.68–2.02)	0.57
	3–4	293	24(8.2)	0.77(0.41-1.46)	0.43	0.67(0.35–1.30)	0.24
Presence of CSVD	None	194	19(9.8)	Ref.		Ref.	
	Presence	692	74(10.7)	1.07(0.63–1.81)	0.80	1.00(0.58–1.71)	0.99
Ischaemic stroke							
Infarction number	NAI	283	8(2.8)	Ref.		Ref.	
	SAI	398	51(12.8)	4.90(2.32–10.32)	<0.001	4.76(2.22–10.22)	<0.001
	MAI	205	37(18.1)	6.94(3.23–14.90)	<0.001	6.65(3.05–14.50)	<0.001
Total CSVD burden	0	194	19(9.8)	Ref.		Ref.	
	1–2	399	50(12.5)	1.24(0.72–2.13)	0.43	1.17(0.68–2.02)	0.57
	3–4	293	24(8.2)	0.77(0.41–1.46)	0.43	0.67(0.35–1.30)	0.24
Presence of CSVD	None	194	19(9.8)	Ref.		Ref.	
	Presence	692	74(10.7)	1.07(0.63–1.81)	0.80	1.00(0.58–1.71)	0.99
CVE							
Infarction number	NAI	283	8(2.8)	Ref.		Ref.	
	SAI	398	51(12.8)	4.90(2.32–10.32)	<0.001	4.76(2.22–10.22)	<0.001
	MAI	205	37(18.1)	6.94(3.23–14.90)	<0.001	6.65(3.05–14.50)	<0.001
Total CSVD burden	0	194	19(9.8)	Ref.		Ref.	
	1–2	399	53(13.3)	1.30(0.76–2.23)	0.33	1.22(0.71–2.10)	0.47
	3–4	293	24(8.2)	0.76(0.40–1.43)	0.39	0.66(0.34–1.26)	0.20
Presence of CSVD	None	194	19(9.8)	Ref.		Ref.	
	Presence	692	77(11.1)	1.10(0.66–1.86)	0.71	1.02(0.60–1.75)	0.93
TIA							
Infarction number	NAI	283	12(4.2)	Ref.		Ref.	
	SAI	398	5(1.3)	0.30(0.11–0.86)	0.025	0.46(0.16–1.35)	0.16
	MAI	205	7(3.4)	0.81(0.32–2.06)	0.660	1.08(0.40–2.91)	0.88
Total CSVD burden	0	194	7(3.6)	Ref.		Ref.	
	1–2	399	10(2.5)	0.65(0.24–1.73)	0.38	0.6899.24–1.96)	0.49
	3–4	293	7(2.4)	0.57(0.19–1.76)	0.33	0.90(0.26–3.07)	0.86
Presence of CSVD	None	194	7(3.6)	Ref.		Ref.	
	Presence	692	17(2.5)	0.62(0.25–1.56)	0.31	0.74(0.28–2.00)	0.56

*CVE*, composite vascular events; *TIA*, transient ischaemic attack; *CSVD*, cerebral small vessel disease; *N.A*., not available; *Ref*, reference.

Model 1: adjusted for age and sex.

Model 2: adjusted for age, gender, body mass index, history of ischaemic stroke, TIA, coronary artery disease, atrial fibrillation, hypertension, diabetes, hypercholesterolaemia, smoking status, time to randomization, qualifying event, NIHSS score on admission, and antiplatelet therapy.

Among 886 included patients, recurrent stroke occurred in 24(25.8%), 50(53.8%), 19(20.4%) patients with severe CSVD, slight CSVD, and non-CSVD, respectively (Table 3), yet after controlling for all potential covariates, no independent associations of CSVD and any recurrence were observed (Table 3). Stroke recurrence rates were no significant difference in patients with or without CSVD no matter receiving clopidogrel plus aspirin or aspirin only (Supplement Table 3). Among patients with different MR phenotypes of CSVD, the risk of stroke recurrence was similar between the clopidogrel plus aspirin group and the aspirin only group (Supplement Table 4).

### Outcome by the status of CSVD and infarction pattern

**Figure 1:**
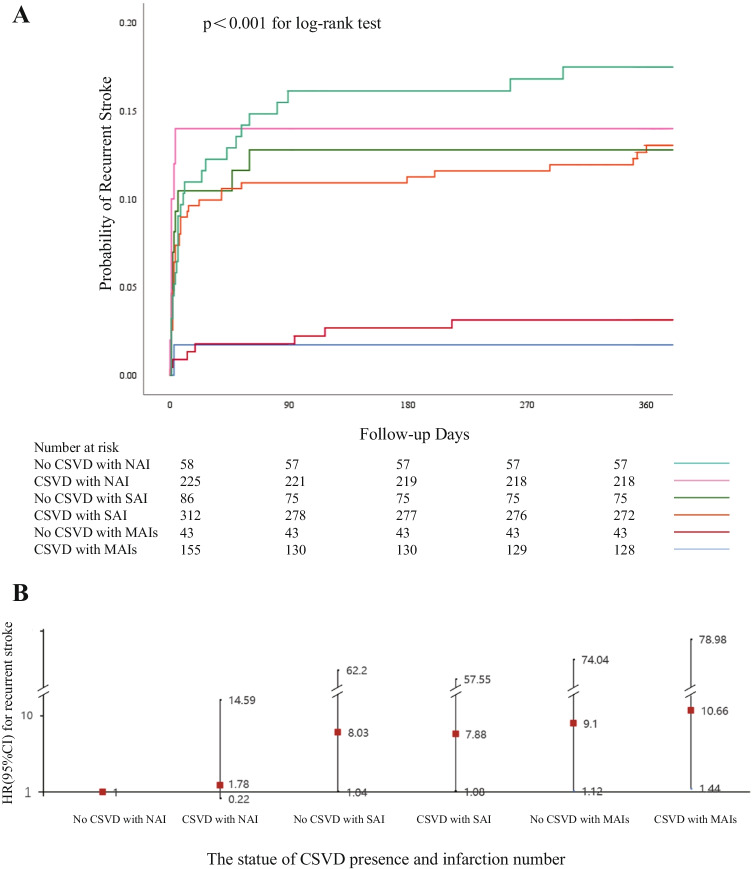
Association of CSVD presence and infarction number with recurrent stroke at 1-year follow-up. **A** Kaplan-Meier curves for probability of recurrent stroke at 1-year follow-up. **B** Cox regressive analysis of predictor for 1-year recurrent stroke based on the status of CSVD presence and infarction number after adjusting age and sex. CSVD, cerebral small vessel disease; NAI, non-acute infarction; SAI, single acute infarction infarction; MAIs, multiple acute infarctions; and HR, hazard ratio.

The risk of clinical events at 1-year follow-up by the status of CSVD presence and infarction number was demonstrated in Table 4. In all 93 patients with recurrent stroke in the 1-year follow-up, recurrent stroke events occurred in 1 (1.1%) patient with non-CSVD and NAI, 7 (7.5%) patients with CSVD and NAI, 11 (11.8%) patients with non-CSVD and SAI, 40 (43.0%) patients with CSVD and SAI, 7 (7.5%) patients with non-CSVD and MAI, and 27 (29.0%) patients with CSVD and MAI. Compared with patients with non-CSVD and NAI, patients with non-CSVD and SAI (HR 8.03, 95% CI from 1.04 to 62.20, *p*=0.05), patients with CSVD and SAI (HR 7.88, 95% CI from 1.08 to 57.55, *p*=0.04), patients with non-CSVD and MAIs (HR 9. 10, 95% CI from 1.12 to 74.04, *p*=0.04), and patients with CSVD and MAIs (HR 10.66, 95% CI from 1.44 to 78.98, *p*=0.02) were all associated with recurrent stroke within 1 year after adjusted for age and sex (Figure 1), and after adjusting for age and sex plus other potential covariates, patients with CSVD and MAI were associated with approximately 9-fold increased risk of recurrent stroke at 1 year (HR 9.56, 95% CI 1.27–71.79, *p*=0.03). Similar results were observed regarding recurrent ischaemic stroke and CVE (Table 4).

**Table 4:** Risk of outcomes by CSVD and infarction number in patients with minor ischaemic stroke or TIA at 1-year follow-up

Outcomes	No.	Event, *n* (%)	Model 1		Model 2	
			Adjusted HR(95%CI)	*p* value	Adjusted HR(95%CI)	*p* value
Stroke						
No CSVD with NAI	58	1(1.7)	Ref.		Ref.	
CSVD with NAI	225	7(3.1)	1.78(0.22–14.59)	0.59	1.73(0.21–14.33)	0.61
No CSVD with SAI	86	11(12.8)	8.03(1.04–62.20)	0.05	7.86(1.00–61.50)	0.05
CSVD with SAI	312	40(12.8)	7.88(1.08–57.55)	0.04	7.27(0.98–54.09)	0.05
No CSVD with MAIs	50	7(14.0)	9.10(1.12–74.04)	0.04	8.86(1.08–72.69)	0.04
CSVD with MAIs	155	27(17.4)	10.66(1.44–78.98)	0.02	9.56(1.27–71.79)	0.03
Ischaemic stroke						
No CSVD with NAI	58	1(1.7)	Ref.		Ref.	
CSVD with NAI	225	7(3.1)	1.78(0.22–14.59)	0.59	1.73(0.21–14.33)	0.61
No CSVD with SAI	86	11(12.8)	8.03(1.04–62.20)	0.05	7.86(1.00–61.50)	0.05
CSVD with SAI	312	40(12.8)	7.88(1.08–57.55)	0.04	7.27(0.98–54.09)	0.05
No CSVD with MAIs	50	7(14.0)	9.10(1.12–74.04)	0.04	8.86(1.08–72.69)	0.04
CSVD with MAIs	155	27(17.4)	10.66(1.44–78.98)	0.02	9.56(1.27–71.79)	0.03
CVE						
No CSVD with NAI	58	1(1.7)	Ref.		Ref.	
CSVD with NAI	225	7(3.1)	1.76(0.22–14.44)	0.60	1.70(0.21–14.06)	0.62
No CSVD with SAI	86	11(12.8)	8.03(1.04–62.17)	0.05	8.00(1.02–62.63)	0.05
CSVD with SAI	312	40(12.8)	7.82(1.07–57.15)	0.04	7.32(0.99–54.42)	0.05
No CSVD with MAIs	50	7(14.0)	9.08(1.12–73.85)	0.04	9.02(1.10–73.93)	0.04
CSVD with MAIs	155	30(19.4)	11.81(1.60–87.17)	0.02	10.84(1.45–81.04)	0.02
TIA						
No CSVD with NAI	58	5(8.6)	Ref.		Ref.	
CSVD with NAI	225	7(3.1)	0.32(0.10–1.07)	0.06	0.36(0.10–1.28)	0.11
No CSVD with SAI	86	1(1.2)	0.13(0.02–1.15)	0.07	0.18(0.02–1.64)	0.18
CSVD with SAI	312	4(1.3)	0.14(0.04–0.53)	0.004	0.24(0.06–1.01)	0.05
No CSVD with MAIs	50	1(2.0)	0.22(0.03–1.93)	0.17	0.30(0.03–2.64)	0.28
CSVD with MAIs	155	6(3.9)	0.40(0.12–1.39)	0.15	0.63(0.17–2.40)	0.50

*CVE*, composite vascular event; *TIA*, transient ischaemic attack; *CSVD*, cerebral small vessel disease; *NAI*, non-acute infarction; *SAI*, single acute infarction; *MAIs*, multiple acute infarctions; *N.A*., not available; Ref, reference.

Model 1: adjusted for age and sex.

Model 2: adjusted for age, gender, body mass index, history of ischaemic stroke, TIA, coronary artery disease, atrial fibrillation, hypertension, diabetes, hypercholesterolaemia, smoking status, time to randomization, qualifying event, NIHSS score on admission, and antiplatelet therapy.

The Kaplan-Meier curves demonstrated that patients with CSVD and MAI had a higher recurrence of stroke during 1-year follow-up than other groups (Table 4; Figure 1; log-rank *p*=0.001). Similar results were also observed for the recurrence of ischaemic stroke and CVE. No significant association between the status of CSVD and infarction number with TIA was found.

### Outcome by the presence of CSVD in different infarction pattern

There was not a significant interaction effect between infarction number and CSVD. Stratified analysis restricted to patients with different infarction number showed that no associations were evident for CSVD and recurrence (Table 5).

**Table 5:** Risk of outcomes by the presence of CSVD when grouped by infarction number in patients with minor ischaemic stroke or TIA at 1-year follow-up

Outcomes	Non-acute infarction (*n*=283)				Single acute infarction (*n*=398)				Multiple acute infarctions (*n*=205)			
	Non-CSVD	CSVD	Adjusted HR (95%CI)	*p* value	Non-CSVD	CSVD	Adjusted HR (95%CI)	*p* value	Non-CSVD	CSVD	Adjusted HR (95%CI)	*p* value
Stroke	1(1.7)	7(3.1)	1.59(0.15–17.28)	0.70	11(12.8)	40(12.8)	0.98(0.48–2.04)	0.96	7(14.0)	27(17.4)	1.03(0.42–2.52)	0.95
Ischaemic stroke	1(1.7)	7(3.1)	1.59(0.15–17.28)	0.70	11(12.8)	40(12.8)	0.98(0.48-2.04)	0.96	7(14.0)	27(17.4)	1.03(0.42–2.52)	0.95
CVE	1(1.7)	7(3.1)	1.59(0.15–17.28)	0.70	11(12.8)	40(12.8)	0.98(0.48–2.04)	0.96	7(14.0)	30(19.4)	1.23(0.46–2.73)	0.79
TIA	5(8.6)	7(3.1)	0.15(0.03–0.71)	0.02	1(1.2)	4(1.3)	0.90(0.06–12.68)	0.94	1(2.0)	6(3.9)	1.68(0.16–18.3)	0.67

*CVE*, composite vascular event; *TIA*, transient ischaemic attack; *CSVD*, cerebral small vessel disease; *N.A*., not available; *Ref*, reference.

Model: adjusted for age, gender, body mass index, history of ischaemic stroke, TIA, coronary artery disease, atrial fibrillation, hypertension, diabetes, hypercholesterolaemia, smoking status, time to randomization, qualifying event, NIHSS score on admission, and antiplatelet therapy.

## Discussion

In the present analysis of the CHANCE clinical trial, we found that the combination of CSVD burden and infarction numbers could increase recurrent stroke risk stratification efficiency after acute minor stroke or TIA within 1 year. The patients with coexistent CSVD and MAIs got the highest risk of new stroke, ischaemic stroke, and CVE than those without CSVD and acute stroke lesion.

Neuroimaging features on baseline, which could provide useful information on the potential mechanisms and physiologies, have drawn much attention as ways to stratify the risk of recurrence in patients with minor stroke and TIA. In this study, we did not find an independent association between CSVD and any recurrent events. It was consistent with the result from the post hoc analysis of the Stenting and Aggressive Medical Management for Preventing Recurrent Stroke in Intracranial Stenosis (SAMMPRIS) study [13]. However, several evidence from previous researches revealed that CSVD was associated with the occurrence and recurrence of ischaemic stroke and TIA in the healthy population and patients with stroke [6, 10, 25–27]. The explanations for these distinct results were as follows. Firstly, the difference in study population and study design may be potential reasons. In our analysis, patients from the CHANCE trial all accepted antiplatelet therapy; however, the risk of recurrent stroke was similar to patients with and without CSVD after dual antiplatelet treatment. Then, multiple comparisons in six groups with different statuses of CSVD and infarction number came with the attendant risk of a type I error. There was a high risk of model overfitting in the smaller subgroups. Moreover, different MR phenotypes of CSVD had different values in predicting ischaemic and hemorrhagic outcomes in patients with MIS or TIA. The overall CSVD burden, which integrated four CSVD imaging markers including WMH, CMBs, lacuna, and ePVS, reflected the cumulative effects of various features of CSVD. Patients receiving dual antiplatelet therapy strategies did not show a lower risk of recurrent stroke than aspirin only in the predominantly ischaemic CSVD subgroup or predominantly micro-hemorrhagic subgroup. Lastly, the visual score for combinations of different neuroimaging markers has been introduced to evaluate the total CSVD burden in this study. These scales are pragmatic but limited insensitivity, and the selected components were given equal weight in the combined score. Neuroimaging features of CSVD were indirect signs of chronic poor cerebral blood flow regulation which could lead to the lacunar syndrome [28]. According to TOAST classification, CSVD is categorized as small perforating arteries disease that arised from the large basal arteries of the brain or their branches and usually resulted in acute lacunar stroke. It provided a more complete overview of the pathological burden in patients with CSVD. In our analysis, due to the limited sample size, the influence of CSVD on stroke outcomes was not explored based on TOAST classification.

Infarction number was also an important neuroimaging parameter for predicting outcomes after acute ischaemic stroke or TIA. In our previous studies of the CHANCE trial, MAIs were related to the highest risk of recurrence than SAI and NAI after acute minor stroke and TIA [5]. This result was supported by a TIA registry org project [8]. Multiple infarctions usually indicated the mechanisms of embolism from the heart, extracranial or intracranial large arteries, and other undetermined sources according to the TOAST classification [5, 29]. MAIs often suggested an underlying embolic source from unstable plaques located in proximal vessels and the heart. In addition, factors simultaneously affecting two or more vessels also resulted in multiple infarctions [30]. Although SAI also could cause by embolism, most single infarction showed only one subcortical infarction on DWI with underlying obstruction of small penetrating brain arteries and arterioles. The mechanism of SAI often indicated atherosclerosis or lipohyalinosis of small perforating arteries and arterioles.

The combination of different neuroimaging characteristics could provide more useful information about the underlying etiology of ischaemic stroke than them only. The burden of CSVD and acute infarction pattern reflected distinct aspects of the potential pathogenesis and pathology of stroke. Although CSVD and MAIs partly overlapped, an important proportion of patients had the presence of CSVD without infarction or the presence of multiple stroke lesions without CSVD (10.0% and 5.6% in the present study, respectively). Infarction number offered information about embolisms, which CSVD represented the chronic cerebral blood flow changes and pathology of arterioles. Reflected the different sides of the mechanism underlying stroke, the presence of both severe CSVD and MAIs indicated a high risk of secondary stroke. ICAS identified by MRA was usually caused by atherosclerotic plaques or thrombus formation and further resulted in cerebral blood flow turbulent and secondary embolism from ruptured plagues and thrombus [31]. Although MAIs and ICAS were both related to a higher risk of recurrent stroke, the simultaneous presence of two neuroimaging parameters had the highest recurrent risk in patients with TIA or minor stroke [12].

Moreover, the combination of imaging signs with traditional predictors also could enhance the risk stratification of recurrent stroke. The traditional ABCD^2^ score, which only based on clinical characteristics (age, blood pressure, clinical features, duration of symptoms, diabetes), was reported to have a modest predictive power for recurrent ischaemic stroke (AUC 0.55 to 0.75) [32]. The ABCD^2^+MRI score (plus diffusion-weighted imaging lesion and vessel occlusion status) and the ABCDE+ score (plus etiology of large-artery atherosclerosis and DWI positivity) increased the predicted ability of recurrence after TIA or acute minor stroke, compared with the traditional ABCD^2^ score [33, 34]. The present studying only provided information about the outcomes of MIS and TIA with different status of CSVD burden and infarction number. We will further explore the prediction of recurrence by combining the CSVD burden with traditional risk factors.

Our study still had several limitations. Firstly, potential selection bias had existed. Only 886 (17.1%) patients with 93 recurrent stroke that were from 45 of 114 participating sites providing MRI were included in the current post hoc analysis. Secondly, only Chinese were included in the CHANCE trial, which may limit the generalizability of the findings to non-Chinese populations. Thirdly, multiple comparisons increase the risk of a type I error as mentioned above. Fourthly, MAIs represented embolism from arteries or other sources, while impaired vascular regulation and blood-brain barrier dysfunction, and thrombus-inflammation may underlie the mechanisms of CSVD. The etiologies of recurrent stroke in patients with different statuses of CSVD and infarction number were not specified in the paper, which were critically important to make sense of the mechanisms. Lastly, given the potential different mechanisms of CSVD subtypes, the associations between ischemic CSVD markers, such as WMH and lacunes, and CMBs and stroke recurrence, were not investigated respectively.

## Conclusion

The coexistent CSVD and MAIs at baseline predicted recurrent stroke in patients with MIS and TIA, indicating the combination of CSVD burden and infarction pattern may improve the effectiveness of risk stratification after acute minor stroke and TIA.

## Supplementary Information

Below is the link to the electronic supplementary material.

Supplementary file1 (DOCX 28 KB)

## Data Availability

The data are available from the corresponding author upon reasonable request.

## References

[1] Wang Y, Li Z, Gu H (2020). China Stroke Statistics 2019: A Report From the National Center for Healthcare Quality Management in Neurological Diseases, China National Clinical Research Center for Neurological Diseases, the Chinese Stroke Association, National Center for Chronic and Non-communicable Disease Control and Prevention, Chinese Center for Disease Control and Prevention and Institute for Global Neuroscience and Stroke Collaborations. Stroke Vasc Neurol.

[2] Yang Y, Zhou M, Zhong X (2018). Dual versus mono antiplatelet therapy for acute non-cardioembolic ischaemic stroke or transient ischaemic attack: a systematic review and meta-analysis. Stroke Vasc Neurol.

[3] Wu CM, McLaughlin K, Lorenzetti DL, Hill MD, Manns BJ, Ghali WA (2007). Early risk of stroke after transient ischemic attack: a systematic review and meta-analysis. Arch Intern Med.

[4] Giles MF, Rothwell PM (2007). Risk of stroke early after transient ischaemic attack: a systematic review and meta-analysis. Lancet Neurol.

[5] Jing J, Meng X, Zhao X (2018). Dual antiplatelet therapy in transient ischemic attack and minor stroke with different infarction patterns: subgroup analysis of the CHANCE randomized clinical trial. Jama Neurol.

[6] Lau KK, Li L, Schulz U (2017). Total small vessel disease score and risk of recurrent stroke. Neurology.

[7] Liu L, Wong KS, Leng X (2015). Dual antiplatelet therapy in stroke and ICAS: subgroup analysis of CHANCE. Neurology.

[8] Amarenco P, Lavallee PC, Labreuche J (2016). One-year risk of stroke after transient ischemic attack or minor stroke. N Engl J Med.

[9] Rensma SP, van Sloten TT, Launer LJ, Stehouwer C (2018). Cerebral small vessel disease and risk of incident stroke, dementia and depression, and all-cause mortality: a systematic review and meta-analysis. Neurosci Biobehav Rev.

[10] Debette S, Schilling S, Duperron MG, Larsson SC, Markus HS (2019). Clinical significance of magnetic resonance imaging markers of vascular brain injury: a systematic review and meta-analysis. Jama Neurol.

[11] Ssi-Yan-Kai G, Nasr N, Faury A (2013). Intracranial artery stenosis or occlusion predicts ischemic recurrence after transient ischemic attack. Am J Neuroradiol.

[12] Pan Y, Meng X, Jing J (2017). Association of multiple infarctions and ICAS with outcomes of minor stroke and TIA. Neurology..

[13] Kwon H, Lynn MJ, Turan TN (2016). Frequency, risk factors, and outcome of coexistent small vessel disease and intracranial arterial stenosis. Jama Neurol.

[14] Chen H, Pan Y, Zong L (2020). Cerebral small vessel disease or intracranial large vessel atherosclerosis may carry different risk for future strokes. Stroke Vasc Neurol.

[15] Mustanoja S, Putaala J, Haapaniemi E, Strbian D, Kaste M, Tatlisumak T (2013). Multiple brain infarcts in young adults: clues for etiologic diagnosis and prognostic impact. Eur J Neurol.

[16] Cho AH, Kim JS, Jeon SB, Kwon SU, Lee DH, Kang DW (2007). Mechanism of multiple infarcts in multiple cerebral circulations on diffusion-weighted imaging. J Neurol.

[17] Regenhardt RW, Das AS, Lo EH, Caplan LR (2018). Advances in understanding the pathophysiology of lacunar stroke: a review. Jama Neurol.

[18] Cuadrado-Godia E, Dwivedi P, Sharma S (2018). Cerebral small vessel disease: a review focusing on pathophysiology, biomarkers, and machine learning strategies. J Stroke.

[19] Wang Y, Wang Y, Zhao X (2013). Clopidogrel with aspirin in acute minor stroke or transient ischemic attack. New Engl J Med.

[20] Wang Y, Johnston SC (2010). Rationale and design of a randomized, double-blind trial comparing the effects of a 3-month clopidogrel-aspirin regimen versus aspirin alone for the treatment of high-risk patients with acute nondisabling cerebrovascular event. Am Heart J.

[21] Wang G, Jing J, Li J, et al. (2020) Association of elevated hs-CRP and multiple infarctions with outcomes of minor stroke or TIA: subgroup analysis of CHANCE randomised clinical trial. Stroke Vasc Neurol 2020–369. 10.1136/svn-2020-000369 PMC8005909 32958697

[22] Amarenco P, Lavallee PC, Labreuche J (2016). One-year risk of stroke after transient ischemic attack or minor stroke. J Vasc Surg.

[23] Wen HM, Lam WW, Rainer T (2004). Multiple acute cerebral infarcts on diffusion-weighted imaging and risk of recurrent stroke. Neurology.

[24] Wardlaw JM, Smith EE, Biessels GJ (2013). Neuroimaging standards for research into small vessel disease and its contribution to ageing and neurodegeneration. Lancet Neurol.

[25] Rensma SP, van Sloten TT, Launer LJ, Stehouwer CDA (2018). Cerebral small vessel disease and risk of incident stroke, dementia and depression, and all-cause mortality: a systematic review and meta-analysis. Neurosci Biobehav Rev.

[26] Staals J, Makin SD, Doubal FN, Dennis MS, Wardlaw JM (2014). Stroke subtype, vascular risk factors, and total MRI brain small-vessel disease burden. Neurology.

[27] Du H, Wilson D, Ambler G (2021). Small vessel disease and ischemic stroke risk during anticoagulation for atrial fibrillation after cerebral ischemia. Stroke.

[28] Østergaard L, Engedal TS, Moreton F (2016). Cerebral small vessel disease: capillary pathways to stroke and cognitive decline. J Cereb Blood Flow Metab.

[29] Bonati LH, Lyrer PA, Wetzel SG, Steck AJ, Engelter ST (2005). Diffusion weighted imaging, apparent diffusion coefficient maps and stroke etiology. J Neurol..

[30] Ay H, Gungor L, Arsava EM (2010). A score to predict early risk of recurrence after ischemic stroke. Neurology.

[31] Jung JM, Kang DW, Yu KH (2012). Predictors of recurrent stroke in patients with symptomatic intracranial arterial stenosis. Stroke.

[32] Chandratheva A, Geraghty OC, Rothwell PM (2011). Poor performance of current prognostic scores for early risk of recurrence after minor stroke. Stroke..

[33] Coutts SB, Eliasziw M, Hill MD (2008). An improved scoring system for identifying patients at high early risk of stroke and functional impairment after an acute transient ischemic attack or minor stroke. Int J Stroke..

[34] Engelter ST, Amort M, Jax F (2012). Optimizing the risk estimation after a transient ischaemic attack - the ABCDE plus sign in circle score. Eur J Neurol..

