# Supplementary material for: Coexistent cerebral small vessel disease and multiple infarctions predict recurrent stroke
Source: Neurol Sci. 2022 Apr 1;43(8):4863–74. doi: 10.1007/s10072-022-06027-6 (PMC9349065; doi:10.1007/s10072-022-06027-6)
Supplement: Supplementary file 1 — Supplementary file1 (DOCX 28 KB) [file 10072_2022_6027_MOESM1_ESM.docx]

**Supplementary Table 1. Baseline characteristics between the included population and the excluded population in CHANCE trail**

| **Characteristics** | **All Patients of**  **CHANCE**  **(n=5170)** | **Patients excluded**  **(n=4284)** | **Patients included**  **(n=886)** | **P value** |
| --- | --- | --- | --- | --- |
| Age, years, median(IQR) | 62.7(54.7-71.3) | 62.6(54.6-71.2) | 62.7(55.2-71.4) | 0.36 |
| BMI, kg/m2, median (IQR) | 24.7(22.7-26.4) | 24.7(22.9-26.5) | 24.4(22.5-26.2) | 0.005 |
| Sex, male(%) | 3420(66.2) | 2830(66.1) | 590(66.6) | 0.76 |
| Alcohol use, n (%) | 1600(31.0) | 1318(30.8) | 282(31.8) | 0.53 |
| Tobacco use, n (%) | 2221(43.0) | 1833(42.8) | 388(43.8) | 0.58 |
| History of disease, n (%) |  |  |  |  |
| Ischaemic stroke | 1033(20.0) | 887(20.7) | 146(16.5) | 0.004 |
| TIA | 174(3.4) | 153(3.6) | 21(2.4) | 0.07 |
| Myocardial infarction | 96(1.9) | 77(1.8) | 19(2.1) | 0.49 |
| Congestive heart failure | 80(1.6) | 64(1.5) | 16(1.8) | 0.49 |
| Atrial fibrillation | 96(1.9) | 75(1.8) | 21(2.4) | 0.21 |
| Diabetes mellitus | 1093(21.1) | 910(21.2) | 183(20.7) | 0.70 |
| Hypertension | 3399(65.7) | 2830(66.1) | 569(64.2) | 0.29 |
| Hyperlipidemia | 573(11.1) | 463(10.8) | 110(12.4) | 0.17 |
| ICAS | 536(49.3) | 426(48.1) | 459(51.9) | 0.11 |
| NIHSS score on admission, median(IQR) | 2(0-2) | 1(0-2) | 2(1-3) | <0.001 |
| Mean time to randomization, hour, median(IQR) | 12(6.5-19.5) | 11.8(6-19.5) | 12.5(7.8-20.0) | <0.001 |
| Qualifying event, n(%) |  |  |  | <0.001 |
| Minor stroke | 3725(72.1) | 2965(69.2) | 760(85.8) |  |
| TIA | 1445(28.0) | 1319(30.8) | 126(14.2) |  |
| Antiplatelet therapy, n(%) |  |  |  | 0.56 |
| Aspirin only | 2586(50.0) | 2135(49.8) | 451(50.9) |  |
| Clopidogrel + aspirin | 2584(50.0) | 2149(50.2) | 435(49.1) |  |
| BMI, body mass index; TIA, transient ischaemic attack; NIHSS, National Institutes of Health Stroke Scale; ICAS, intracranial atherosclerotic stenosis. | | | | |

**Supplementary Table 2. Baseline characteristics between include population and the excluded population in the imaging substudy of CHANCE trail**

| **Characteristics** | **All Patients of the imaging substudy**  **(n=1089)** | **Patients excluded**  **(n=203)** | **Patients included**  **(n=886)** | **P value** |
| --- | --- | --- | --- | --- |
| Age, years, median(IQR) | 63.1(55.2-71.6) | 65.0(55.8-72.7) | 62.7(55.2-71.4) | 0.10 |
| BMI, kg/m2, median (IQR) | 24.3(22.5-26.2) | 24.2(22.5-26.1) | 24.4(22.5-26.2) | 0.93 |
| Sex, male(%) | 713(65.6) | 123(60.6) | 590(66.7) | 0.10 |
| Alcohol use, n (%) | 335(30.8) | 53(26.1) | 282(31.8) | 0.11 |
| Tobacco use, n (%) | 458(42.1) | 70(34.5) | 388(43.8) | 0.02 |
| History of disease, n (%) |  |  |  |  |
| Ischaemic stroke | 187(17.2) | 41(20.2) | 146(16.5) | 0.21 |
| TIA | 32(2.9) | 11(5.4) | 21(2.4) | 0.02 |
| Myocardial infarction | 19(1.7) | 0(0) | 19(2.1) | 0.04 |
| Congestive heart failure | 19(1.7) | 3(1.5) | 16(1.8) | 0.75 |
| Atrial fibrillation | 21(1.9) | 0(0) | 21(2.4) | 0.03 |
| Diabetes mellitus | 227(20.8) | 44(21.7) | 183(20.7) | 0.75 |
| Hypertension | 710(65.2) | 141(69.5) | 569(64.2) | 0.16 |
| Hyperlipidemia | 137(12.6) | 27(13.3) | 110(12.4) | 0.73 |
| ICAS | 551(50.7) | 92(45.5) | 459(51.9) | 0.11 |
| NIHSS score on admission, median(IQR) | 2(0-2) | 0(0-2) | 2(1-3) | <0.001 |
| Mean time to randomization, hour, median(IQR) | 12.0(7.2-19.5) | 10.0(6.0-17.1) | 12.5(7.8-20.0) | <0.001 |
| Qualifying event, N(%) |  |  |  | <0.001 |
| Minor stroke | 824(75.7) | 64(31.5) | 760(85.8) |  |
| TIA | 265(24.3) | 139(68.5) | 126(14.2) |  |
| Antiplatelet therapy, n(%) |  |  |  | 0.64 |
| Aspirin only | 558(51.2) | 107(52.7) | 451(50.9) |  |
| Clopidogrel+aspirin | 531(48.8) | 96(47.3) | 435(49.1) |  |
| BMI, body mass index; TIA, transient ischaemic attack; NIHSS, National Institutes of Health Stroke Scale; ICAS, intracranial atherosclerotic stenosis. | | | | |

**Supplement Table 3. Effect of clopidogrel plus aspirin compared with aspirin only on outcomes in patients with/without CSVD**

| **Outcomes** |  | **Non-CSVD(n=194)** | | | | **CSVD(n=692)** | | | |
| --- | --- | --- | --- | --- | --- | --- | --- | --- | --- |
|  |  | **Aspirin only No.(%)** | **Clopidogrel plus aspirin No.(%)** | **Adjusted HR(95%CI)** | **P value** | **Aspirin only No.(%)** | **Clopidogrel plus aspirin No.(%)** | **Adjusted HR(95%CI)** | **P value** |
| Stroke |  | 10(10.5) | 9(9.1) | 0.48(0.17-1.34) | 0.16 | 40(11.2) | 34(10.1) | 0.86(0.55-1.37) | 0.53 |
| Ischaemic stroke |  | 10(10.5) | 9(9.1) | 0.48(0.17-1.34) | 0.16 | 40(11.2) | 34(10.1) | 0.86(0.55-1.37) | 0.53 |
| CVE |  | 10(10.5) | 9(9.1) | 0.48(0.17-1.34) | 0.16 | 42(11.8)) | 35(10.4 | 0.85(0.54-1.32) | 0.47 |
| TIA |  | 6(6.3) | 1(1.0) | 0.02(0.001-0.66) | 0.03 | 7(2.0) | 10(3.0) | 1.59(0.58-4.32) | 0.37 |
| CVE, composite vascular events; TIA, transient ischaemic attack; CSVD, cerebral small vessel disease; N.A., not available; Ref, reference.  Model: adjusted for age, gender, body mass index, history of ischaemic stroke, TIA, coronary artery disease, atrial fibrillation, hypertension, diabetes, hypercholesterolaemia, smoking status, time to randomization, qualifying event, NIHSS score on admission, and antiplatelet therapy. | | | | | | | | | |
|  | | | | | | | | | |

**Supplement Table 4. Effect of clopidogrel plus aspirin compared with aspirin only on outcomes in patients with different MR phenotypes of CSVD**

| **Outcomes** | **Ischaemic CSVD^1^** | | | | **Ischaemic CSVD^2^** | | | | **Microhaemorhagic CSVD^3^** | | | |
| --- | --- | --- | --- | --- | --- | --- | --- | --- | --- | --- | --- | --- |
|  | **Aspirin only No.(%)** | **Clopidogrel +Aspirin No.(%)** | **Adjusted HR(95%CI)** | **P value** | **Aspirin only No.(%)** | **Clopidogrel +Aspirin No.(%)** | **Adjusted HR(95%CI)** | **P value** | **Aspirin only No.(%)** | **Clopidogrel +Aspirin No.(%)** | **Adjusted HR(95%CI)** | **P value** |
| Stroke | 10(10.5) | 9(9.1) | 0.48(0.17-1.33) | 0.16 | 28(13.7) | 22(11.3) | 0.80(0.45-1.41) | 0.44 | 12(7.9) | 12(8.5) | 0.85(0.36-1.98) | 0.85 |
| Ischaemic stroke | 10(10.5) | 9(9.1) | 0.48(0.17-1.34) | 0.16 | 28(13.7) | 22(11.3) | 0.80(0.45-1.41) | 0.44 | 12(7.9) | 12(8.5) | 0.85(0.36-1.98) | 0.85 |
| CVE | 10(10.5) | 9(9.1) | 0.48(0.17-1.34) | 0.16 | 30(14.7) | 23(11.8) | 0.77(0.45-1.34) | 0.36 | 12(7.9) | 12(8.5) | 0.50(0.09-2.83) | 0.50 |
| TIA | 6(6.3) | 1(1.0) | 0.02(0.001-0.66) | 0.03 | 3(1.5) | 7(3.6) | 2.94(0.70-12.34) | 0.14 | 4(2.6) | 3(2.1) | 1.01(0.95-1.08) | 0.77 |
| CVE, composite vascular events; TIA, transient ischaemic attack; CSVD, cerebral small vessel disease; WMH, white matter hyperintensity; PVS, perivascular space; CMB, cerebral microbleed; N.A., not available; Ref, reference.  1：the presence of WMH, lacuna and/or PVS; 2: the presence of WMH and lacuna; 3: the presence of CMBs  Model: adjusted for age, gender, body mass index, history of ischaemic stroke, TIA, coronary artery disease, atrial fibrillation, hypertension, diabetes, hypercholesterolaemia, smoking status, time to randomization, qualifying event, NIHSS score on admission, and antiplatelet therapy. | | | | | | | | | | | | |
